# Overview of the practices of on-farm emergency slaughter of cattle in the Nordic countries

**DOI:** 10.1186/s13028-022-00627-0

**Published:** 2022-04-04

**Authors:** Gíslína Skúladóttir, Clare Joan Phythian, Ingrid Hunter Holmøy, Guro Myhrene, Karin Alvåsen, Adam Dunstan Martin

**Affiliations:** 1 Department of Production Animal Clinical Sciences, Faculty of Veterinary Medicine, Norwegian University of Life Sciences, Postboks 5003 NMBU, 1432 Ås, Norway; 2 Section for Small Ruminant Research, Faculty of Veterinary Medicine, Norwegian University of Life Sciences, Svebastadveien 112, 4325 Sandnes, Norway; 3 Norwegian Food Safety Authority, Jærveien 12, 4319 Sandnes, Norway; 4 Department of Clinical Sciences, Faculty of Veterinary Medicine and Animal Science, Swedish University of Agricultural Sciences, PO Box 7054, 75007 Uppsala, Sweden

**Keywords:** Animal hygiene, Cattle, Mortality, On farm emergency slaughter, Slaughter

## Abstract

On farm mortality is an increasing problem in cattle production systems in the Nordic countries. It represents an economic loss to the farmer and raises questions of sustainability, food waste and animal welfare. On-farm emergency slaughter (OFES) represents, in some situations, an opportunity for a farmer to salvage some of the economic value from an animal that cannot be transported to a slaughterhouse. The basis of the regulation of OFES in the Nordic countries originates largely from legislation from the European Union. However, this review has found that the availability and practice of OFES in the Nordic countries differs considerably. For example, in Norway 4.2% of all cattle slaughter is OFES, whilst in Iceland OFES has never been recorded. National food safety authorities have issued differing regulations and guidelines regarding the suitability of sick and injured animals for OFES. This review shows there is a paucity of data regarding the incidence and reasons for the use of OFES of cattle in the Nordic countries and points out the need for more investigation into this area to improve veterinary education, consumer protection and animal welfare.

## Background

On farm mortality encompasses all livestock death on farm including unassisted deaths, euthanasia, slaughter for home consumption, and on farm emergency slaughter (OFES) for commercial purposes. A definition of the terms used in this article to describe the death of an animal can be found in Table 1. Incidence of on farm mortality is an animal welfare indicator whereby high levels of mortality are associated with poor animal welfare [1, 2]. In addition to raising concerns about animal welfare, high levels of on farm mortality damage the reputation of the cattle industry with the public and raise significant questions on the sustainability of cattle production systems [3, 4]. The death of animals on farm also leads to significant economic loss in the form of lost sales value, loss of production, cadaver disposal costs, and increased herd replacement costs [5, 6]. Despite advances in animal husbandry and veterinary medicine the incidence of bovine on farm mortality in the Nordic countries (Denmark, Finland, Iceland, Norway and Sweden), and worldwide, has steadily increased over the past 30 years [2, 3, 7]. The reasons for the increase in incidence of on farm mortality are multifactorial and have yet to be fully elucidated [3, 5, 6, 8]. However, it has been postulated in Sweden [7] and Denmark [9] that part of the increases seen could be due, at least in part, to changes in legislation regarding the transport [10] and slaughter [11] of cattle in the European Economic Area (EEA).

**Table 1:** Definitions of terms associated with mortality used in this article

Term	Definition
On-farm mortality	The death of an animal on farm, irrespective of the manner in which it died. Home slaughter, euthanasia, OFES as well as unassisted/uncontrolled death
Home slaughter	Animal is slaughtered on farm without a veterinary *ante-mortem* inspection. Meat may be used domestically—but sale is prohibited
On-farm emergency slaughter (OFES)	Animal is slaughtered on farm having passed an *ante-mortem* veterinary inspection. The carcass is transported to a slaughterhouse whereby it undergoes a *post-mortem* inspection. Sale of the meat allowed
Casualty slaughter	The slaughter of a sick or injured animal at a slaughterhouse. The animal undergoes normal *ante-mortem* and *post-mortem* controls. Sale of meat allowed
Euthanasia	The killing of an animal with no intention of human consumption

The minimum standards for food safety and animal health and welfare within the EEA are regulated by European legislation which is binding throughout the European Union (EU) and are adapted into national legislation by EEA countries that are outside of the EU. Of the Nordic countries, Denmark, Finland, and Sweden are members of the EU, whilst Iceland and Norway are members of the EEA. Home slaughter of animals for consumption, not sale, is allowed without any attestation throughout Europe. However, the slaughter of animals for sale is tightly regulated in the EEA. In 2004, the European Council decided that only healthy animals which have been slaughtered at a slaughterhouse are eligible for human consumption [11]*.* To ensure compliance all animals slaughtered for human consumption, and subsequent sale, must undergo an *ante-mortem* inspection by a veterinary surgeon in the 24 h preceding slaughter, as described in Section III of Annex II of Regulation (EC) 853/2004 [11] and legislated for in Regulation (EU) 2017/625 [12]. Usually, this is achieved by an Official Veterinarian examining the animals upon arrival to, or whilst being held in lairage at, a slaughterhouse. Guidelines for *ante-mortem* inspections have been developed [13].

Despite legislation prohibiting the slaughter of sick and injured animals for human consumption, provided strict criteria are met, animals which are injured or suffering from an aliment which does not endanger food safety can be slaughtered [11]. If the criteria are met the animal should be transported to a slaughterhouse for slaughter providing the transport will not cause additional suffering [10]. The slaughter of ill or injured animals at a slaughterhouse is termed casualty slaughter. However, ill or injured animals are generally considered unfit for transport [10]. In this situation the legislation allows for OFES if specific criteria are met as described in Chapter VI of Annex III of Regulation (EC) 853/2004 [11]. Clear definitions of ‘slightly ill or injured’ as well as ‘additional suffering’ are absent from the European regulations [14].

The OFES of ungulates is permitted provided the slaughtered animal is; *an otherwise healthy animal [that] must have suffered an accident that prevented its transport to the slaughterhouse for welfare reasons* [11]. In order to process animals killed on farm and intended for human consumption, slaughterhouses must have in place facilities to receive and process OFES [12]. An *ante-mortem* examination is still required for animals that undergo OFES. Specific exceptions from the requirement that *ante-mortem* inspection is performed in a slaughterhouse, Article 18 (2) (a) of Section II of Regulation (EU) 2017/625 [12], is made in Article 4 of Regulations (EU) 2017/625. Article 4 allows for an *ante-mortem* inspection by an official veterinarian to be performed outside of the slaughterhouse subject to compliance with the requirements for emergency slaughter laid down in points (1), (2) and (6) of Chapter VI of Section I of Annex III to Regulation (EC) No 853/2004 [11, 12].

There is evidence that there is within country variation regarding the practice of transport and slaughter regulations which increases stress on stakeholders [9, 15–17]. The lack of unequivocal definitions for key terms such as, ‘slightly ill or injured’, ‘additional suffering’, ‘accident’ in European legislation [10, 11, 14] means that practice in individual countries is likely to vary. There are considerable cultural and migratory ties between the Nordic countries, including veterinarians crossing borders to work and study. However, the extent of harmonisation of the practice of OFES between the Nordic countries is unknown. The aim of this article is to summarise the legislation and practice of OFES in the Nordic countries.

## Material and methods

This article is an overview review article [18] and as such does not aim to provide an exhaustive review of research that has previously been carried out. The review of the legislation, recommendations, practices, and literature was initially performed in December 2020 and January 2021. A follow up review of the literature, legislation and national guidelines occurred in the first week of January 2022 to account for any changes in practice and legislation introduced since the initial review.

### Literature search

A PubMed search (https://pubmed.ncbi.nlm.nih.gov/) using the combined terms (((("On farm emergency slaughter") OR ("casualty slaughter") OR ("emergency slaughter"))) AND (cow OR cattle OR bovine)) NOT (spongiform) was used to gain an overview of published scientific work which linked with the focus area of this article. The term ‘spongiform’ was excluded from the literature search to remove articles dealing primarily with the control of transmissible spongiform encephalopathies (TSEs). This is because animals suffering from TSEs are not eligible for OFES in Europe.

### Search for statistics on population and slaughter

Data on the cattle population and numbers of animals slaughtered annually were provided by searching the European Commission's official statistics body—Eurostat (www. ec.europa.eu/eurostat). Additional searches were performed to cross- check and complete missing data with national interest bodies (Landbrug & Fødevarer—the Danish Agriculture and Food Council, Luke—the National Resources Institute Finland, Bændasamtök Íslands—The Icelandic Farmers Association, Animalia—The Norwegian Meat Research Centre, Jordbruksverket—the Swedish Agricultural Board). Where data on the numbers of cattle which underwent OFES were not available from European and national statistics, the competent authority in each country was contacted by email to ascertain if records of the number of OFES were kept. The competent authorities for emergency slaughter in the Nordic countries are; the Danish Veterinary and Food Authority (www.foedevarestyrelsen.dk), the Finnish Food Authority (www.ruokavirasto.fi), the Icelandic Food and Veterinary Body (www.mast.is), the Norwegian Food Safety Authority (www.mattilsynet.no), and the Swedish Food Agency (www.livsmedelsverket.se).

### Search for legislation and national guidelines

In addition to the literature reviewed the authors accessed the relevant European Council decisions pertaining to OFES in the EEA [10, 11, 19]. Further an internet search of the competent authorities for food safety in each of the Nordic countries was performed to ascertain the legislation, and the availability of guidelines for the OFES of cattle in each country, respectively. Further each competent authority was contacted by email and asked to describe their OFES regulations and the extent of the practice in their country. The practices in each country were summarised on a country-by-country basis before being compared. Specific guidelines provided by the competent bodies regarding conditions in which OFES was/was not appropriate was summarised in a table (Table 3).

## Results

### Literature review

The described PubMed search identified 39 documents, 24 of which were from the year 2000 or later. Of these 24 documents 6 were from Canada and 18 from European countries. Seven of the documents originating from Europe were opinion letters written to the scientific journal Veterinary Record. Of the remaining 11 documents originating in Europe two are best characterised as case studies which resulted in emergency slaughter, one dealt with medicine residues, and one with animal welfare from birth until slaughter. Three articles from Ireland reported the investigation of the reasons for casualty slaughter, it’s certification and practice, two articles from Italy dealt with the reasons for on farm death and how culling can be managed, and a Spanish article dealt with culling in herds using robotic milking machines. No literature was found concerning OFES, or casualty slaughter, in the Nordic countries. Furthermore, no literature was found comparing practices between countries, which highlights the need for further knowledge in this area.

### National cattle population and numbers slaughtered

Denmark and Sweden have the largest cattle populations of the Nordic countries with around 1.5 million head of cattle in each country. Finland and Norway both have a cattle population of around 850,000. Iceland has a smaller population, around 80,000. The numbers of cattle reported to be slaughtered annually in each country broadly correlates with the national cattle population, with each country slaughtering between 29 and 35% of its cattle population annually. More than 98% of cattle known to be slaughtered in Denmark, Finland and Sweden were slaughtered in slaughterhouses, compared to 95% in Norway, and 83% in Iceland. Domestic slaughter was highly prevalent in Iceland, whilst OFES was the predominant form of slaughter outside of a slaughterhouse in Norway. Table 2 summarizes the details of the cattle population, the number of animals slaughtered and location of slaughter for each of the Nordic countries.

**Table 2:** Details of the cattle population and numbers of animals slaughtered in the Nordic countries in 2019

	Cattle population	Adult cows	Dairy cows	Beef Cows	Known number of cattle slaughtered	Number of cattle slaughtered in a slaughterhouse (% of known slaughtered)	Number of cattle slaughtered for domestic consumption (% of known slaughtered)	Number of OFES cattle (% of known slaughtered)
Denmark	1,500,000^a^	645,800^a^	563,000^a^	83,000^a^	468,000^c,d^	464,000 (99.1%)^c^	4000 (0.9%)^d^	Unknown
Finland	840,420^a^	318,360^a^	258,940	59,420	247,000^c,d^	242,940 (98.4%)^c^	4060 (1.6%)^d^	Unknown
Iceland	80,900 ^a^	29,000^a^	26,200 ^a^	2900 ^a^	27,130^c,d^	22,730 (83.8%)^c^	4400 (16.2%)^d^	None
Norway	862,550^b^	307,484^b^	215,069^b^	92,415^b^	304,953^b^	292,153 (95.8%)^b^	Unknown	12,800 (4.2%)^b^
Sweden	1,404,670^a^	499,700^a^	301,380^a^	198,320^a^	441,780 ^c,d^	432,770 (98.0%) ^c^	9010 (2.0%)^d^	Unknown

Data sources:

^a^Anonymous. Bovine population—annual data 2019. European Commission’s official statistics body—Eurostat. https://ec.europa.eu/eurostat/databrowser/view/APRO_MT_LSCATL__custom_697055/default/table?lang=en. Accessed 17 March 2022

^b^Anonymous. The status of meat production 2019. *In Norwegian*. 2020. Animalia. https://www.animalia.no/contentassets/3dce35cde68a47b091097fa8c6ec2dd5/kjottets-tilstand-2019.pdf. Accessed 17 March 2022

^c^Anonymous. Estimates of slaughtering, in slaughterhouses—annual data 2019. European Commision's official statistics body – Eurostat. https://ec.europa.eu/eurostat/databrowser/view/APRO_MT_PANN/default/table?lang=en&category=agr.apro.apro_anip.apro_mt.apro_mt_p. Accessed 17 March 2022

^d^Anonymous. Estimates of slaughtering, other than in slaughterhouses—annual data 2019. European Commission’s official statistics body – Eurostat. https://ec.europa.eu/eurostat/databrowser/view/apro_mt_sloth/default/table?lang=en. Accessed 17 March 2022

### General requirements for OFES in European Legislation

The OFES of ungulates for sale and human consumption is permitted provided the slaughtered animal is; *an otherwise healthy animal* [that] *must have suffered an accident that prevented its transport to the slaughterhouse for welfare reasons* [11]. This definition was first made in Chapter VI of Annex III of the Council Regulation (EC) No 853/2004 [11], and is referred to in Regulation (EU) 2017/625 [19].

According to European regulations the following (paraphrased) criteria must be met for animals slaughtered on farm to be processed and passed as fit for human consumption [11, 19]:

1. A veterinarian must carry out an *ante-mortem* inspection of the animal.
2. The animal, killed and bled, must be transported hygienically to the slaughterhouse, without delay. Removal of stomach and intestines is allowed under veterinarian supervision, on-site, but all parts removed must follow to the slaughterhouse, identified to the right carcass.
3. If transport takes over two hours, the carcass must be refrigerated, although not actively if climate conditions allow.
4. A declaration by the farmer of the identity of the animal and medication and withdrawal periods, must accompany the animal to the slaughterhouse.
5. A declaration issued by the veterinarian recording the favourable outcome of the *ante-mortem* inspection, the date and time of, and reason for the emergency slaughter, and details of any recent treatments, must accompany the slaughtered animal to the slaughterhouse.
6. That the carcass is deemed fit for human consumption after *post-mortem* inspection.
7. That the slaughterhouse follows the instructions given by the veterinarian of use of meat.

### Denmark

The competent body monitoring OFES in Denamrk is the Danish Veterinary and Food Administration (DVFA). The Danish translation of the EU regulations states the first three requirements as in the EU regulation; namely that the animal is healthy and has suffered an accident, that an *ante-mortem* inspection must be performed and that the animal must be killed, bled and transported to a slaughterhouse as soon as possible [11]. A sick animal cannot be slaughtered and sold, but the owner can decide if he thinks it is fit for consumption and perform slaughter for home consumption. According to the DVFA all slaughterhouses are equipped to receive OFES, although it is mostly the smaller slaughterhouses that do accept them (Jacob Gade, DVFA, personal communication).

The DVFA published a guide for farmers for OFES. The guide states that the animal owner must call a veterinarian for the *ante-mortem* inspection as a requisite for human consumption of the meat, and that it is the veterinarian’s responsibility to decide if the animal is fit for human consumption. The second requirement is that the owner fills out a declaration including details on the animal to be slaughtered, the veterinary drugs the animal has received in the previous six months and a description of the accident that led to the OFES [20]. The DVFA provides a list of examples of what acceptable circumstances for OFES are. These examples include fractures and calving associated lesions, and a fresh wound in the hoof, e.g., puncture by a nail. The DVFA guidelines state that a cow with hypocalcaemia (milk fever) that could be treated, or an abscess in the hoof, should not be slaughtered as OFES [21]. Further DVFA guidelines state that cattle that can’t be handled safely during transport due to their temperament are appropriate for OFES [21].

The veterinarian’s role is to perform an *ante-mortem* inspection and confirm that the animal is fit for human consumption. The veterinarian also has a responsibility to describe clinical findings and the reason it may not be transported to a slaughterhouse. The veterinarian is also required to describe any treatment that has been given to the animal with the withdrawal period and comment on the ‘accident’ that resulted in OFES. The veterinarian can perform the slaughter, in which case they attest for this and the date and time of slaughter. Alternatively, the veterinarian can state the time in which the animal needs to be killed by a slaughterman before transportation. If the animal is killed after the deadline set by the veterinarian the animal will not be deemed fit for human consumption, even if seen by the veterinarian in the 24 h preceding slaughter.

### Finland

The competent body monitoring OFES in Finland is the Finnish Food Authority (FFA). The FFA regards the practice of OFES in Finland as rare, stating that home slaughter is more frequent, as it is simpler. Many slaughterhouses (or farmers) do not have a proper hygienic vehicle for transporting a carcass to a slaughterhouse. Furthermore, there may be difficulties in finding a veterinarian fast enough to perform the *ante-mortem* inspection (R. McLean, personal communication). Despite this the FFA have produced a guide which is primarily aimed at veterinarians working with meat inspection frequently which describes well the legal framework and requirements for OFES, linking clearly to the relevant European regulations [22]. In addition, guidelines are provided as to which animals may or may not be suitable for emergency slaughter [22].

The Finnish legislation follows the European legislation and the eight criteria that must be fulfilled in the European legislation to allow for OFES are all mentioned in the Finnish guidelines. The animal must have suffered an ‘accident’ to be eligible for OFES. The term accident is broadened to accept accidents, falls and ruptures in the 24 h preceding slaughter. The FFA gives examples of animals eligible for OFES. These include an animal; which has slipped and suffered a sprain in the past 24 h, or has a broken limb, a large wound, or a traumatised teat. The guidelines then specify several conditions that are not eligible for OFES because they do not result from an accident. These include animal’s that have suffered from milk fever, dislocation of the abomasum, uterine prolapse, or acute mastitis. Furthermore, the guidelines state that animals which have been recumbent for more than 24 h, are ineligible for OFES.

Certification from the producer and veterinarian are required to accompany the carcass to the slaughterhouse. The producer must certify the animal’s identity, the date, and details of any treatments (veterinary or otherwise) the animal has received, and any withdrawal periods for the medicinal treatments received. The veterinarian needs to certify the reason for the OFES, the result of the *ante-mortem* inspection, and the date and time of killing. According to the Finnish guidelines the veterinarian is required to confirm that slaughter was performed in an appropriate manner and confirm the time of slaughter. Consequently the veterinarian must be present during the stunning and exsanguination [22].

### Iceland

The competent body for OFES in Iceland is Icelandic Food and Veterinary Body (IFVB). In 2012 Iceland included the regulations in Chapter 7 Article 15 of Council Regulation (EC) 853/2004 into Icelandic law [23]. However, no updates in this legislation have occurred since 2012. On-farm emergency slaughter is defined as; *“when an animal is killed outside a slaughterhouse, according to a veterinarian’s decision, because of an accident or other reasons and the animal is then taken to slaughter in a slaughterhouse and its products used for human consumption"*. The specified requirements of *ante-mortem* inspection, killing, bleeding and transport, mimic those in Council Regulation (EC) No 853/2004. A declaration by the veterinarian who performed the *ante-mortem* inspection is to follow the carcass to the slaughterhouse. It is to include the reason for OFES, and detail any medicines given to the animal in the last month of the animal’s life. The slaughterhouse veterinarian is required to perform a *post-mortem* examination, and ensure the viscera were removed within three hours after the stunning and exsanguination and perform a microbiological testing of the product [23].

Despite the regulations allowing OFES in Iceland the practice has yet to be performed. Currently there are no slaughterhouses equipped to receive OFES and as such the IFVB has not issued a form to be used in the case of OFES, or any guidelines on the practice.

### Norway

The competent body monitoring OFES in Norway is the Norwegian Food Safety Authority (NFSA). The European legislation regarding OFES has been translated and accepted in Norwegian national legislation with one important difference; the term ‘accident’ has been translated to ‘unforeseen event’. Whilst it follows that the definition of accident in the English language is ‘an unforeseen incident, usually with negative effects’ [14], the term probably allows for a slightly wider interpretation than is available in the original European legislation. The NFSA has published guidelines on the application of the OFES regulations. These emphasize the requirement for an animal to have been subjected to an accident or unforeseen event which means that the animal is not allowed to be transported to a slaughterhouse, whilst the general condition of the animal is not affected in a way which prevents human consumption of the meat [24].

The NFSA guidelines for OFES specifically state that injuries sustained during calving, are eligible for OFES providing the animal to be slaughtered is not suffering from one or more of the of the following: infection, uterine torsion, mutation, or something similar to the three examples provided. The guidance further states that a prolapse is acceptable as a reason for OFES if the general condition of the animal is unaffected. The same applies for lame and recumbent cattle (providing under 24 h of recumbency when killed). Traumatic accidents, such as fractures and wounds, are also listed as an appropriate reason for OFES, and the guidelines point out the need for almost immediate slaughter in these cases. Post-partum first-calf cows which cannot be milked due to their temperament are eligible for OFES in the first week post-partum [24].

The NFSA guidelines specifically advise against the use of OFES in certain cases. These include mastitis, displacement of the abomasum, and cattle with a wild temperament. The guidelines emphasize that OFES should occur as soon as possible after the accident, with the only exception being grade 2–3 lameness on the 5-point scale, as described by Sprecher et al. [25]. Those cattle can be treated for up to a week after the first injury and undergo OFES if they have not sufficiently improved within seven days. The guidelines also allow for OFES of cattle that have previously had milk fever, that at the time of *the ante-mortem* inspection show no clinical signs of the disorder apart from recumbency providing slaughter occurs within 24 h of the first sign of the disorder.

The NFSA has published a form which has to accompany carcasses to the slaughterhouse (https://www.mattilsynet.no/skjema/nodslakteattest.1678/binary/N%C3%B8dslakteattest). The form requires details on the holding the animal is from, as well as the animals signalment (including date of birth and ear-tag number). Further a description of the accident/unforeseen event which has resulted in the emergency slaughter as well as a statement about the animal’s general state of health is required. The farmer must also attest for the medicines the animal has been treated with in the preceding 30 days as well as treatment with any other medicine with a withdrawal period greater than 30 days. The veterinarian is required to sign the following declaration: *‘I have not found or been made aware of conditions that would make this animal unsuitable for human consumption (alternative euthanasia and destruction)*. The veterinarian then has a space in which he or she can make any comments they feel appropriate. The certificate is then signed, and the time and date of the signature recorded. A final box is for the slaughterman to complete which just states the time and date of death with space for any comments. Currently, the veterinarian performing the *ante-mortem* inspection needs no further training beyond their veterinary degree. However, the NFSA will soon require that veterinarians performing *ante-mortem* inspection have undertaken an additional training course to allow them to perform these OFES *ante-mortem* inspections as an ‘official veterinarian’. All the slaughterhouses in Norway which slaughter cattle offer OFES as a service.

### Sweden

The competent body monitoring OFES in Sweden is the Swedish Food Agency (SFA). The practice of OFES is uncommon in Sweden, although around 30 small-scale slaughterhouses offer this service. Slaughter for home consumption of animals is possible, but these carcasses may only be consumed in the producers’ own household. Mobile slaughterhouses have been commercially available, but this practice was only used to a very small extent [8, 26]. Official written guidelines from the SFA on the practice of OFES are unavailable.

In order for OFES to occur in Sweden an official veterinarian must perform an *ante-mortem* examination and complete a form produced by the SFA (https://www.livsmedelsverket.se/globalassets/produktion-handel-kontroll/blanketter/livs_071_2013_01_veterinarintyg-vid-nodslakt.pdf). The form does not require the farmer to complete or certify any information. The veterinarian must; (i) identify the animal and its location, (ii) identify the slaughterhouse to which the animal will be transported, (iii) describe the animal’s condition, including the reason for OFES and any treatment the animal has received. The veterinarian is required to declare that an otherwise healthy animal suffered an accident that prevents its transport to the slaughterhouse and state the time and date of *ante-mortem* examination. Further the veterinarian needs to certify that the records and documents associated with the animal are legally correct and do not constitute an obstacle to slaughter. The last section of the form requires information on the time and date of stunning and exsanguination certified by an authorised slaughterman.

### Specific guidelines relating to clinical conditions

Three of the five Nordic countries (Denmark, Finland and Norway) provide guidelines for how OFES should be practiced. These include examples of clinical conditions that are, and are not, acceptable for OFES which are summarized in Table 3.

**Table 3:** The guidelines provided by the competent authorities in Denmark, Finland and Norway regarding the acceptability of different clinical conditions for on-farm emergency slaughter

	Denmark	Finland	Norway
Trauma less than 24 h old, e.g. splits at calving, broken bone	✓	✓	✓
Mastitis	✘	✘	✘
Milk fever	✘	✘	✓ ^*^
Uterine prolapse	**–**	✘	✓
Displaced abomasum	**–**	✘	✘
Chronic lame	✘	✘	✘
Wild—dangerous to handle	✓	✘	✘

Key: ‘✓’ acceptable for OFES, ‘✘’ unacceptable for OFES, ‘**–** ‘ condition not mentioned in guidelines

^*Cows with clinical milk fever are not acceptable for OFES^

## Discussion

The practice of OFES varies throughout the Nordic countries. Iceland has no record of an animal been slaughtered in this way whilst in Norway 4.2% of all the cattle slaughtered in 2019 were OFES. Interestingly the inter-country differences in the number of cattle slaughtered on-farm for human consumption become greater when the estimates of cattle slaughtered on farm for home consumption and OFES are combined. In this situation the estimates for the proportion of animals slaughtered for human consumption become 0.9%, 1.6%, 16.2%, 4.2%, 2.0%, for Denmark, Finland, Iceland, Norway and Sweden, respectively. Whilst this article has highlighted some differences in the practice and guidelines surrounding OFES, the legislative framework for OFES is almost identical, meaning that other factors must account for these differences.

High levels of on farm mortality are not compatible with sustainable agricultural practices [2, 3, 8]. Despite this and the increased focus on animal’s welfare on farm mortality in cattle production systems has been increasing [2, 3]. Whilst reducing the incidence of on farm mortality should be a priority for animal welfare and economic reasons there will always be deaths on farms. On farm emergency slaughter, and slaughter for home consumption, represent ways to mitigate food waste. In Norway 7% of dairy cows died on Norwegian dairy farms in 2019, almost half of these animals (44%) underwent OFES [27]. Similar on farm mortality statistics have been presented for the Danish and Swedish dairy industries [7, 8, 28], and there is little reason to believe the figures would be hugely different in Finland. However, in contrast to Norway very few of the animals dying on farms in the EU Nordic countries (Denmark, Finland and Sweden) are salvaged for human consumption. The number of animals undergoing OFES in these three countries is virtually negligible and proportionally very few animals undergo home slaughter. Although in this regard a greater proportion of Swedish cattle are salvaged by home slaughter than is the case for Danish or Finnish cattle.

Studies have shown that a reasonable estimate for on farm mortality amongst dairy cows in Denmark, Finland, and Sweden is 6.5% [3, 6, 7, 27]. Using this estimate approximately 73,000 of the 1,123 million, dairy cows in these countries die on farm annually (Table 2). If 40% of these carcasses could be salvaged for human consumption (44% are salvaged in Norway) this would represent approximately 29,000 cows. In 2019, 17,160 animals in the EU Nordic countries were estimated to be home slaughtered (Table 2). If it is assumed all of these were dairy cows, so as to not overestimate, that would result in at least an estimated 12,000 dairy cows which were potentially fit for human consumption were destroyed in 2019. This unrefined estimate makes broad generalizations about the causes of on farm mortality between countries. For example, it is assumed that the reasons for on farm mortality, and the potential to salvage meat from the animals that died are the same between the Nordic countries. Further this estimate assumes that there is the possibility to harmonize the regulations throughout the Nordic countries and that there is equal access to OFES, which is not currently the case. Despite these limitations they illustrate a large potential to salvage meat from animals that died on-farm.

The legislation for OFES in the Nordic countries is virtually identical. Despite this the practice differs considerably. National guidelines regarding the eligibility of animals suffering from specific clinical conditions for OFES have been published by the competent authorities in Demark, Finland and Norway and are summarized in Table 3. In Denmark the guidelines specifically allow for wild cattle to undergo OFES, whilst the guidelines in Norway specifically prohibit this, and whilst wild animals are not mentioned specifically in the Finnish guidelines, they fall outside of the guidelines. Interestingly cows suffering from a uterine prolapse are specifically mentioned as been eligible for OFES in Norway but are ineligible in Finland, where the Finnish guidelines specifically state that a uterine prolapse does not constitute an accident. Finnish guidelines state that a cow that has gotten milk fever, is not eligible for OFES, for the same reason as prolapse, while the Norwegian one state that if the animal has been treated for milk fever, that arose in the last 24 h, is now without clinical signs of the disorder, but recumbent, they are eligible. Perhaps most importantly from an animal welfare perspective, lame animals are suitable for OFES in Norway according to the guidelines published by the NFSA. The same guidelines also dictate that one can try and treat low grade lameness for up to a week, before deciding on OFES. However, the same animal would fall outside of the guidelines in Denmark and Finalnd. As lameness is perhaps the greatest single welfare problem in cattle production systems [29] it is vitally important that an overview is gathered regarding the outcomes of lame animals so that appropriate steps can be taken to improve their welfare.

National differences exist in the certification and slaughter requirements between the Nordic countries. The example certificates published on the national competent bodies all vary somewhat, this is despite an example certificate now been available in Chapter 5, Annex IV of the Commission implementing regulation (EU) 2020/2235 [30] being made available to facilitate harmonization of practices. Responsibility for the identification of the animal to be slaughtered and listing of previous treatments varies between the countries. In Finland, Norway and Denmark this is the producer’s responsibility, whilst in Sweden this responsibility lies with the veterinarian.

All countries require the *ante-mortem* inspection of the animal to be slaughtered within the 24 h preceding its death. In Denmark the certificating veterinarian can reduce the time interval from *ante-mortem* inspection to slaughter, whilst this is not possible in Norway. Having the ability to reduce the time from *ante-mortem* inspection to slaughter potentially both enhances animal welfare and protects public health. In Finland the veterinarian must see the killing, whilst in all other countries this can be delegated to a third qualified person. Which may be one of the reasons there are so few OFES in Finland? Both Denmark and Norway require the veterinarian to fill in the time and date of *ante-mortem* inspection, while in Finland it is enough to fill in the time and date of stunning, as the veterinarian must oversee that action.

Despite having the possibility in law, OFES is scarcely practiced in four of the five Nordic countries. Norway and Iceland are members of the EEA, but not the EU, which affords the countries a greater degree of self-determination over agricultural policy than EU member states have, both in terms of legislative practice and economic policy. Iceland, however, has no tradition of OFES, and no facilities for it, meaning it is very hard to practice while following the legislation of meat hygiene. The situation in Finland is similar and the country currently lacks the infrastructure which would allow for OFES to be commonplace.

The Nordic countries all have high labour costs, compared to other European countries [31]. This means that labour intensive procedures, such as travelling long distances to salvage meat quickly, become uneconomical if beef is traded freely in an internal European market [32]. Sweden, for example, had a tradition for OFES [7]. However, since the 1990’s the costs associated with this procedure have led to a situation where most of the injured animals are euthanized on farm and sent to a destruction plant*.* In Norway there are considerable market support mechanisms which mean that beef is priced above the international market value which perhaps contributes to the large numbers of OFES in the country [32]. One of the four aims of Norwegian agricultural policy is to maintain agriculture throughout the entire country [33]. This, combined with the fact that farmer owned cooperatives dominate cattle and meat production in Norway, means that financial support mechanisms are in place to facilitate OFES, which has a long tradition in the country. Changing attitudes towards sustainability and may mean that it might be appropriate in the future to evaluate the cost–benefit calculations associated with OFES in a broader context than simply the finances of the procedure. Animal welfare, the environmental impact and the minimization of food waste, are all factors which society are increasingly attaching importance in a wider debate about the sustainability of food production and these issues are closely linked to OFES.

Meat harvesting is strictly controlled to protect the consumer from food borne disease and animals from unnecessary suffering. Clinically sick and injured animals pose a higher risk, at least theoretically, to the consumer than healthy animals slaughtered in the slaughterhouse. The shedding of zoonotic pathogens, such as enterotoxigenic *Escherichia coli*, are known to increase in stressed animals [34, 35]. It is also likely that in many cases animals may be dirtier than they might otherwise be when killed on farm because, for example, they are recumbent at the time of slaughter, or that the carcass of the animal is handled sub-optimally after killing. Concerns about food safety and meat quality have led to 89% of veterinarians working in slaughterhouses in the Republic of Ireland not wanting to accept OFES carcasses despite the practice been legal [36]. A Canadian study into the perceptions of OFES found that a significant proportion of stakeholders had concerns about OFES reducing food safety compared to regular slaughter [16].

On-farm emergency slaughter represents an exception to these regulations which benefits the primary producer. The consumer is, however, most likely unaware of the practice. Stakeholders are typically divided as to whether the public perception of the dairy industry would be enhanced or damaged if the public became aware of OFES [16]. Swift and effective use of OFES could reduce undue suffering, particularly in the case of genuine accidents, by offering a primary producer the possibility to salvage some of the value of an animal by acting swiftly and performing OFES. It also helps prevent unassisted on farm mortality by reducing financial loss [8]. However, stakeholders have identified that the existence of OFES may mean that animals which could be preventatively culled due to, for example ‘poor feet’, may be rebred as there is always the possibility of OFES for animals if they become lame [16]. Other challenges to animal welfare identified were if producers choose to wait for medicine withdrawal periods before performing OFES, as opposed to immediately euthanizing the animal [16]. Current guidelines in Norway specifying that lame cattle can be treated for up to a week before OFES are a well-intentioned balance between ‘salvaging meat’ and preserving animal welfare by limiting the number of days animal can be lame before slaughter. However, they risk producers not contacting veterinarians until later in a disease process to preserve the option of OFES for longer.

It is worth noting that animals undergoing OFES all have suboptimal animal welfare. On farm mortality, which includes OFES, is one of the measures used in the welfare assessment protocols used throughout Europe, ‘Welfare Quality’ [37]. Therefore, it is surprising how few data are available about OFES [17, 38]. Even in Norway, which has the most comprehensive and available statistics on OFES, there is no information available about the reasons OFES was performed on cattle, and the extent to which the practice falls within, or outside, of the national interpretation of the legislation. If the processes around OFES, and slaughter for home consumption, are to be understood there is a need to identify the reasons animals undergo on farm slaughter and the decision-making processes around the practice. The availability of data regarding OFES would allow for genuine comparisons and evidence-based decisions to be made when evaluating practices with and between countries. The differences in the guidelines issued by the Norwegian, Finnish, and Danish Food Safety Authorities clearly demonstrates that practices are not harmonized within the EEA, despite harmonized legislation.

The differences in implementation and practices despite having near identical legislation pose problems for the consumer, who believes, an EU/EEA health marked product is produced according to identical guidelines and practices. It further poses considerable problems for veterinary practitioners who increasingly practice in different countries [39] with different traditions regarding OFES. Veterinary surgeons in Europe and North America have highlighted frailties in the operational efficiency of OFES caused by the conflict of interests of a producer’s own veterinarian deciding on the eligibility of an animal for OFES [15, 16]. The differing practices between countries shows that universities and official veterinarians should teach in such a way that learners understand that identical legislation can be practiced in very different ways depending on the national interpretation of legislation. Ultimately this level of education can only be provided if there is more concrete data available regarding the reasons for, use of, and practices associated with OFES.

## Conclusions

This review has demonstrated that despite harmonised legislation in the Nordic countries practice of OFES differs considerably. There is a lack of knowledge about the reasons for the national differences in the practicing of OFES as well as the reasons why animals undergo OFES. These knowledge gaps require further investigation.

## Data Availability

The datasets used and/or analyzed during the current study are available from the corresponding author on reasonable request.

## Abbreviations

DFSA: Danish Food and Safety Authority
EEA: European Economic Area
EU: European Union
FFA: Finnish Food Authority
NFSA: Norwegian Food and Safety Authority
OFES: On Farm Emergency Slaughter
SFA: Swedish Food Authority
